# Supplementary material for: Humoral and Cellular Immune Response in Asymptomatic Dogs with Visceral Leishmaniasis: A Review
Source: Vaccines (Basel). 2022 Jun 14;10(6):947. doi: 10.3390/vaccines10060947 (PMC9229064; doi:10.3390/vaccines10060947)
Supplement: Supplementary file 1 [file vaccines-10-00947-s001.zip › vaccines-1690212-supplementary.pdf]

Table S1. Keywords used for systematic searches in the Medline database (PubMed).

General Search Terms *	Search Terms Referring to the Clinical Status of Dogs	Search Terms Referring to a Parasite-Specific Immune Response
Visceral leishmaniasis;<br>*Leishmania donovani*;<br>*Leishmania chagasi*;<br>*Leishmania infantum*	AND asymptomatic dog<br>AND susceptible dog<br>AND subclinical disease AND dogs	AND Immune response<br>AND Immune system<br>AND humoral<br>AND cellular response<br>AND Th1 response<br>AND Th2 response<br>AND cytokine

* Each one of these keywords was searched along with each cluster of terms in the following columns.

Table S2. Keywords used in the secondary literature review in the Medline database (PubMed).

General Search Terms	Search Terms Referring to the Clinical Status of Dogs	Search Terms Added in the Secondary Review Referring to:					
		Phenotypic Characterization		Functional Characterization			Humoral Response
		T- and B-Cell Markers	Activation Status of T Cells	Cytokine Profile	NO and ROS Production	Proliferative Capacity	IgG Subtype
Visceral leishmaniasis;<br>*Leishmania donovani*;<br>*Leishmania chagasi*;<br>*Leishmania infantum*	AND asymptomatic dog;<br>AND susceptible dog;<br>AND subclinical disease AND dogs	CD5<br>CD3<br>CD4<br>CD8<br>Foxp3<br>T regulatory cell;<br>Treg<br>CD21	CD45RB<br>CD45RA<br>MHC-II<br>Activated T cells<br>Activation of T cells	IL-4<br>IL-12<br>IL-2<br>IL-6<br>IFN-γ<br>TNF-α<br>IL-10<br>IL-18<br>TGF-β<br>IL-17	Nitric oxide;<br>Oxidative stress;<br>Reactive oxygen species	Lymphoproliferative	IgG1<br>IgG2

NO: Nitric oxide; ROS: Reactive oxygen species; Treg: regulatory T cells.

Table S3. Studies that analyze the lymphoproliferative response of PBMC upon *Leishmania* antigens from infected and control dogs.

Antigens Used			Clinical Classification (Number of Dogs)	Geographical Location	Type of Infection	Lymphoproliferative Response (PR)	Reference
Common Antigens Used		Additional Antigens Used					
**SLA**	10 μg/mL	-	AD ($n = 6$)<br>SD ($n = 7$)<br>CD	Barajas (Spain)	Experimental	PR upon SLA in PBMC from AD, but not in SD.	[1]
		-	AD ($n = 7$)<br>SD ($n = 3$)<br>CD	Portugal	Natural & Experimental	PR upon SLA in PBMC from both SD and AD.	[2]
		-	AD ($n = 78$)<br>OD ($n = 2$)<br>PD ($n = 7$)	Madrid (Spain)	Natural	PR upon SLA in 27% of AD dogs, but in none of SD dogs.	[3]
		P-8	AD ($n = 8$)<br>OD ($n = 12$)<br>PD ($n = 1$)<br>CD ($n = 9$)	Cali (Colombia) and Madrid (Spain)	Experimental	PR upon SLA and P-8 in PBMC from AD and OD, but not in SD.	[4]
		HSP-70, PFR-2, KMP-11	AD ($n = 4$)<br>OD ($n = 5$)<br>PD ($n = 4$)<br>CD ($n = 2$)	Spain	Experimental	PR upon SLA in PBMC from AD, but not in SD. PR upon HSP-70 and PFR-2 in PBMC was moderate.	[5]
	20 μg/mL	rLeIF, rLdp23	AD ($n = 7$)<br>SD ($n = 10$)<br>CD ($n = 10$)	Naples area and Reggio Emilia (Italy)	Natural	PR upon rLeIF and rLdp23, but not SLA, only in PBMC from AD dogs.	[6]
		-	AD ($n = 2$)<br>SD ($n = 4$)<br>CD ($n = 20$)	St Feliu de Codines (Spain)	Experimental	PR upon SLA in PBMC from both AD and SD.	[7]
		-	AD ($n = 132$)<br>SD ($n = 106$)<br>CD ($n = 20$)	Barcelona and Mallorca (Spain)	Natural	PR upon SLA in PBMC from some AD dogs.	[8]
	25 μg/mL	f/t *L.c.* antigen	AD	Cali (Colombia)	Experimental	PR upon f/t *L.c.* antigen was	[9]

			OD<br>PD<br>CD			similar and low in PBMC from all groups.	
**AM**	1x10^7 amastigotes	rLdccys1	AD (*n* = 18)<br>OD (*n* = 14)<br>SD (*n* = 17)<br>CD (*n* = 6)	Teresina (Brazil)	Natural	PR upon AM and rLdccys1 in PBMC from AD, but low or negative in OD and SD.	[10]
**f/t *L.i.* antigen**	1 μg/mL	gp63	AD (*n* = 5)<br>SD (*n* = 11)<br>CD (*n* = 3)	Kemisset and Rabat (Morocco)	Natural & Experimental	PR upon f/t *L.i.* antigen and gp63 in PBMC from AD, but not in SD.	[11]
	5 μg/mL	rCPA, rCPB	AD (*n* = 7)<br>SD (*n* = 4)<br>CD (*n* = 3)	Meshkinshahr (Iran)	Natural	PR upon all antigens in PBMC from AD, but low or negative in SD.	[12]
		rCPA, rCPB, CTE	AD (*n* = 7)<br>SD (*n* = 6)<br>CD (*n* = 3)	Meshkinshahr (Iran)	Natural	PR upon all antigens in PBMC from AD, but not in SD.	[13]
	10 μg/mL	-	Infected resistant (*n* = 4)<br>Infected susceptible (*n* = 3)<br>Clinical (*n* = 4)<br>CD (*n* = 2)	Iowa (USA)	Natural	PR upon f/t *L.i.* antigen in PBMC $CD4^+$ T cells from infected resistant and susceptible dogs, but low in clinical dogs.	[14]
		-	AD<br>SD<br>(*n* > 18)	Iowa (USA)	Natural	PR upon f/t *L.i.* antigen in $CD4^+$ and $CD8^+$ T cells from AD, but not in SD.	[15]
	50 μg/mL	-	AD (*n* = 37)<br>CD (*n* = 34)	Oporto (Portugal)	Natural	PR upon f/t *L.i.* antigen in 5% of AD dogs, but none in CD dogs.	[16]
		-	AD (*n* = 49)<br>CD (*n* = 25)	South of Alijo (Portugal)	Natural	PR upon f/t *L.i.* antigen in 41% of AD dogs, but none in CD dogs.	[17]

PR: proliferative response; PBMC: peripheral blood mononuclear cells; f/t antigen: freezed/thawed antigen; AM: amastigote extracts; SLA: soluble *Leishmania* antigen; AD: asymptomatic dogs; OD: oligosymptomatic dogs; SD: symptomatic dogs; PD: polysymptomatic; CD: control healthy dogs; *L.i.*: *Leishmania infantum*; *L.c.*: *Leishmania chagasi*.

Table S4. Studies that analyze the cytokine production profile of infected and control dogs.

Cytokine Profile	Cytokines Evaluated	Detection Technique	Clinical Classification (Number of Dogs)	Geographical Location	Type of Infection	Antigen Stimulation (PBMC/ PB /Serum)	Main Findings †	Reference
**Th1**	IFN-γ	CPE assay	AD (*n* = 6)<br>SD (*n* = 7)<br>CD (-)	Barajas (Spain)	Experimental	SLA (PBMC)	IFN-y activity only in AD.	[18]
**Th2**	IL-4, IL-10	FC	AD (*n* = 7)<br>SD (*n* = 7)<br>CD (*n* = 7)	USA and Natal (Brazil)	Natural	Unstimulated (PBMC)	↑ % $IL10^+$ B cells in SD (***) and AD (*) *versus* CD.<br>↑ % IL-4$^+$ IgD^{hi} B cells in SD *versus* CD (*).	[19]
**Mixed Th1/Th2 profile**	IFN-γ, IL-4	FC	AD (*n* = 23)<br>SD (*n* = 22)<br>CD (*n* = 30)	Campania region (Italy)	Natural	Unstimulated (PBMC)	↑ % IFN-γ^+ IL-4$^-$ T cells in infected dogs *versus* CD (*).	[20]
			AD-I (*n* = 34)<br>AD-II(*n* = 20)<br>OD (*n* = 8)<br>SD (*n* = 42)<br>CD (*n* = 28)	Belo Horizonte (Brazil)	Natural	SLA (PB)	↑ % IFN-γ^+ $CD4^+$, IFN-γ^+, and IL-4$^+$ $CD8^+$ T cells in AD-II and SD *versus* AD-I and CD (****).<br>↑ % IL-4$^+$ $CD4^+$ T cells in SD *versus* CD (***).	[21]
			AD (*n* = 20)<br>SD (*n* = 20)<br>CD (*n* = 20)	Atenas (Greece)	Natural	SLA (PB)	↑ % IFN-γ^+ and IL-4$^+$ $CD4^+$ and $CD8^+$ T cells in SD *versus* AD and CD.	[22]
	IFN-γ, IL-10	ELISA	Infected resistant (*n* = 4)<br>Infected suscep-	Iowa (USA)	Natural	f/t *L.i.* antigen (PBMC)	↑ IFN-γ in subclinical and resistant dogs *versus* clinical dogs and	[14]

		tible (n = 3)<br>Clinical (n = 4)<br>CD (n = 2)				CD (*).<br>↑ IL-10 with disease progression (*).	
	FC and ELISA	AD<br>SD<br>(n > 18)	Iowa (USA)	Natural	f/t *L.i.* antigen (PBMC)	↑ IL-10 $^{+}$ CD4 $^{+}$ T cells in SD *versus* AD (**).<br>↓ IFN-γ $^{+}$ CD4 $^{+}$ (*) and CD8 $^{+}$ (ns) T cells in SD *versus* AD.	[15]
	ELISA	State I (n = 10)<br>State IIa (n = 27)<br>State Iib (n = 9)<br>State III (n = 10)<br>State IV (n = 4)	Catalunya and Baleares (Spain)	Natural	SLA (PB)	↑ IFN-γ in stage I and Iia dogs *versus* stage Iib, III, and IV (*).<br>Similar levels of IL-10 between groups.	[23]
IFN-γ, IL-4, IL-10	ELISA	AD (n = 18)<br>OD (n = 14)<br>SD (n = 17)<br>CD (n = 6)	Teresina (Brazil)	Natural	rLdccys (PBMC)	↑ IFN-γ in AD and OD.<br>↑ IL-4 only in SD.<br>↑ IL-10 in SD and OD.	[10]
	ELISA and RT-qPCR	AD<br>OD<br>PD<br>CD	Cali (Colombia)	Experimental	SLA (PBMC)	↑ IFN-γ in PD *versus* AD and OD (*).<br>Similar levels of IL-10 between groups.	[9]
IFN-γ, TNF-α, IL-4, IL-10	RT-qPCR	AD (n = 13)<br>SD (n = 9)	Apulian region (Italy)	Natural	SLA (PBMC)	↑ IFN-γ, TNF-α, IL-4, and IL-10 in SD *versus* AD (*).	[24]
IFN-γ, TNF-α, IL-4, IL-10, IL-18	RT-qPCR	AD (n = 8)<br>OD (n = 12)<br>PD (n = 1)<br>CD (n = 9)	Cali (Colombia) and Madrid (Spain)	Experimental	SLA and P-8 (PBMC)	↑ IFN-γ and TNF-α in AD *versus* PD.<br>Similar levels of IL-4 between groups.<br>↓ IL-10 in AD and OD.<br>↑ IL-18 in PD.	[5]
		AD (n = 4)<br>OD (n = 5)<br>PD (n = 4)<br>CD (n = 2)	Spain	Experimental	SLA, HSP-70, PFR-2 and KMP-11 (PBMC)	↑ IFN-γ and TNF-α in AD *versus* SD.<br>Similar levels of IL-4 between groups.	[5]
IFN-γ, TNF-α, IL-4, IL-10, IL-18, IL-2, IL-6	Semiquantitative RT-PCR	AD (n = 7)<br>CD (n = 5)	Unknown	Experimental	SLA (PBMC)	↑ IFN-γ (*), IL-4 (*), IL-10 (ns), and IL-2 (ns) in AD *versus* CD.	[25]
IFN-γ, TNF-α, IL-10, IL-18, IL-2, IL-6, IL-7, IL-8, IL-15	ELISA and Milliplex Map Kit	AD (n = 22)<br>Stage I (n = 21)<br>Stage III (n = 15)	Camaçari (Brazil)	Natural	Unstimulated (serum)	↑ IL-10 in dogs with stage I and II (ns).<br>↑ IL-18 and IL-6 in AD (ns).	[26]
	Luminex assay	AD (n = 20)<br>CD (n = 8)	NE areas of Brazil	Natural & Experimental	Unstimulated (serum)	↑ IFN-γ, IL-10, IL-18, and IL-6 in AD *versus* CD (***).<br>↓ TNF-α, IL-2, and IL-8 in AD *versus* CD (***).<br>Similar levels of IL-7 and IL-15 between groups.	[27]
IFN-γ, IL-4, IL-2, IL-12	RT-qPCR	AD (n = 13)<br>SD (n = 29)<br>CD (n = 11)<br>TD (n = 11)	E areas from Portugal and Brazil	Natural	Unstimulated (PB)	↑ IFN-γ, IL-4, IL-2, and IL-12 in SD *versus* CD (*).<br>↑ IL-4 in AD *versus* CD (*).	[28]
IFN-γ, IL-4, IL-10, IL-18, IL-2, IL-12, IL-18	RT-qPCR	AD (n = 20)<br>ADD (n = 20)<br>SD (n = 20)<br>CD (n = 20)	Campania (Italia)	Natural	Unstimulated (PB)	↑ IFN-γ, IL-4, IL-10, IL-18, and IL-2 in AD.<br>↑ IL-18 in ADD and, later in the infection, ↑ all cytokines analyzed.<br>↑ Th1 and Th2 cytokines in SD.	[29]

CPE: cytopathic effect inhibition assay; FC: flow cytometry; RT-qPCR: quantitative reverse transcription polymerase chain reaction; PBMC: peripheral blood mononuclear cells; PB: peripheral blood; f/t antigen: freezed/thawed antigen; SLA: soluble *leishmania* antigen; AD: asymptomatic dogs; AD-I; asymptomatic dogs with negative serology; AD-II: asymptomatic dogs with positive serology; ADD: asymptomatic dogs who progressed overt the disease; OD: oligosymptomatic dogs; SD: symptomatic dogs; PD: polysymptomatic dogs; CD: control healthy dogs; TD: treated infected dogs; E: endemic areas; NE: non-endemic areas; *L.i.*: *Leishmania infantum*; *L.c.*: *Leishmania*

chagasi. † These results refer to cytokine production after stimulation with *Leishmania* antigens, in those articles where PBMC/peripheral blood stimulation is carried out. $p < 0.05$ (*), $p < 0.01$ (**), $p < 0.001$ (***), $p < 0.0001$ (****), and not significant (ns).

Table S5. Studies that analyze the humoral response in infected and control dogs.

Isotype Analyzed	Subclass Analyzed	Detection Technique	Antigen (Type of Antibody)	Clinical Classification (Number of Dogs)	Geographical Location	Type of Infection	Main Findings	Reference
IgG	IgG1, IgG2	ELISA	*L.i.* antigens (polyclonal)	AD (*n* = 6)<br>TSD (*n* = 8)<br>CD (*n* = 22)	France	Natural	IgG2 associated with AD.	[30]
			L.i. antigens (polyclonal)	AD (*n*= 1)<br>OD (*n* = 1)<br>SD (*n* = 2)<br>CD (*n* = 1)	Caceres (Spain)	Experimental	IgG1 associated with SD.	[31]
			L.i. sonicated promastigotes (polyclonal)	AD (*n* = 11)<br>SD (*n* = 139)<br>TSD (*n* = 25)<br>TD (*n* = 6)	Priorat and Barcelona (Spain)	Natural & Experimental	IgG1 associated with SD.	[32]
			SLA (polyclonal)	AD (*n* = 7)<br>SD (*n* = 3)<br>CD	Portugal	Natural & Experimental	Similar IgG1 levels among groups.<br>IgG2 associated with SD.	[2]
			SLA (polyclonal)	AD<br>SD<br>CD	Alto Douro region (Portugal)	Natural	IgG2 associated with SD.	[33]
			f/t *L.i.* antigen, rCPA, rCPB, and CTE (polyclonal)	AD (*n* = 7)<br>SD (*n* = 6)<br>CD (*n* = 3)	Meshkinshahr (Iran)	Natural	IgG2 associated with AD.	[13]
			f/t *L.i.* antigen (polyclonal)	AD (*n* = 13<br>SD (*n* = 42)<br>CDE (*n* = 21)<br>CDNE (*n* = 18)	E and NE areas from Brazil	Natural	IgG2 associated with SD.	[34]
			Protein A (polyclonal)	AD (*n* = 29)<br>SD (*n* = 23)<br>CDE (*n* = 3)<br>CDNE (*n* = 19)	Spain and France	Natural	IgG1 associated with SD.<br>↑ IgG2 in AD and SD.	[35]
			SLA (polyclonal)	AD (*n* = 12)<br>OD (*n* = 12)<br>SD (*n* = 16)<br>CD (*n* = 20)	Minas Gerais (Brazil)	Natural	IgG1 associated with AD.<br>IgG2 associated with SD.	[36]
			P-8 and SLA (polyclonal)	AD (*n* = 8)<br>OD (*n* = 12)<br>PD (*n* = 1)<br>CD (*n* = 9)	Cali (Colombia) and Madrid (Spain)	Experimental	IgG1 associated with SD.	[4]
			L.i. antigen (polyclonal)	AD (*n* = 2)<br>SD (*n* = 4)<br>CD (*n* = 20)	St Feliu de Codines (Spain)	Experimental	IgG1 associated with SD.	[7]
			SLA (polyclonal)	AD (*n* = 116)<br>SD (*n* = 72)	Alto Douro (Portugal)	Natural	IgG2 associated with SD.	[37]
			SLA (polyclonal)	AD (*n* = 7)<br>OD (*n* = 22)<br>SD (*n* = 13)	Belo Horizonte (Brazil)	Natural	Similar IgG1 levels among groups.<br>IgG2 associated with SD.	[38]
			L.i. antigen (polyclonal)	AD (*n* = 24)<br>SD (*n* = 21)<br>CDNE (*n* = 24)<br>CDE (*n* = 64)	Spain	Natural	Similar IgG1 levels among groups.<br>IgG2 associated with SD.	[39]
			SLA (polyclonal)	AD<br>OD<br>PD<br>CD	Cali (Colombia)	Experimental	Similar IgG1 and IgG2 levels among groups.	[9]
			CLA (monoclonal)	AD (*n* = 37)<br>SD (*n* = 22)	Brazil	Natural	IgG1 and IgG2 ↑ in SD.	[40]
			f/t *L.i.* antigen (polyclonal)	Infected resistant (*n* = 4)	Iowa (USA)	Natural	IgG1 and IgG2 ↑ in susceptible infected and clinical	[14]

				Infected susceptible (*n* = 3)<br>Clinical (*n* = 4)<br>CD (*n* = 2)			groups.	
			SLA (polyclonal)	AD (*n* = 20)<br>SD (*n* = 20)<br>CD (*n* = 5)	Belo Horizonte (Brazil)	Natural	IgG1 associated with AD.<br>IgG2 associated with SD.	[41]
			SLA (polyclonal)	AD-I (*n* = 8)<br>AD-II (*n* = 10)<br>SD (*n* = 16)<br>CD (*n* = 7)	Belo Horizonte (Brazil)	Natural	Low IgG1 levels in all groups.<br>IgG2 ↑ in AD-II and SD.	[42]
			L.c. and *L.b.* antigens (polyclonal)	AD (*n* = 12)<br>SD (*n* = 25)<br>CD (*n* = 17)	Rio de Janeiro (Brazil)	Natural	IgG1 and IgG2 ↑ in SD.	[43]
			SLA (polyclonal)	AD (*n* = 10)<br>SD (*n* = 68)<br>CD (*n* = 7)	Fortaleza (Brazil)	Natural	IgG1 associated with AD.<br>IgG2 associated with SD.	[44]
			CLA or SLA (polyclonal)	AD^{+} (*n* = 21)<br>AD^{-} (*n* = 62)<br>SD^{+} (*n* = 52)<br>SD^{-} (*n* = 25)	Araçatuba (Brazil)	Natural	IgG1 associated with SD.	[45]
			SLA (polyclonal)	AD (*n*= 28)<br>SD (*n* = 22)	Vila Santana do Cafezal (Brazil)	Natural	IgG1 associated with SD.<br>IgG2 high in both AD and SD.	[46]
			SLA, LACK & LeIF (polyclonal)	AD (*n* = 26)<br>SD (*n* = 34)<br>CD (*n* = 26)<br>CDE (*n* = 45)	Tunisia	Natural	IgG1 and IgG2 ↑ in SD.	[47]
			LiAg or A2 protein (polyclonal)	AD (*n* = 10)<br>SD (*n* = 10)<br>VD (*n* = 12)<br>CD (*n* = 12)	Barra Mansa (Brazil)	Natural	IgG1 associated with SD.<br>IgG2 associated with AD.	[48]
			Portions of r-pot B domain (polyclonal)	AD (*n* = 11)<br>OD(*n* = 12)<br>SD (*n* = 15)<br>CD (*n* = 10)<br>CDE (*n* = 17)	Minas Gerais (Brazil)	Natural	IgG1 and IgG2 associated with AD.	[49]
		Western Blot	*L.i.* antigens (polyclonal)	AD (*n* = 78)<br>OD (*n* = 2)<br>PD (*n* = 7)	Madrid (Spain)	Natural	IgG2 associated with SD.	[3]
			Promastigote extract (polyclonal)	AD (*n* = 9)<br>SD (*n* = 5)<br>CD (*n* = 52)	Attiki area (Greece)	Natural	IgG2:IgG1 ratio ↑ in AD *versus* SD.	[50]
	IgG1, IgG2, IgG3, IgG4	ELISA	CLA (monoclonal)	AD<br>OD<br>PD<br>(*n*= 126)	Salvaterra (Brazil)	Natural	IgG1, IgG2, IgG3, and IgG4 associated with SD.	[51]
			CLA (monoclonal)	AD (*n* = 120)<br>SD (*n* = 24)<br>VD (*n* = 40)	Belo Horizonte (Brazil)	Natural	IgG1 and IgG4 associated with AD.<br>IgG2 associated with SD.<br>Low IgG3 levels in AD and SD.	[52]
			L.c. lysate (polyclonal and monoclonal)	AD (*n* = 45)<br>SD (*n* = 45)<br>VD (*n* = 37)<br>CDNE (*n* = 45)	Sao Paolo (Brazil)	Natural	Using pAb, IgG1 and IgG2 ↑ in AD and SD.<br>Using mAb, IgG1, IgG3 and IgG4 ↑ in SD.	[53]
**IgM**	-	ELISA	SLA (polyclonal)	AD<br>SD<br>CD	Alto Douro region (Portugal)	Natural	Low IgM values in infected dogs.	[33]
			SLA (polyclonal)	AD (*n* = 12)<br>OD (*n* = 12)<br>SD (*n* = 16)<br>CD (*n* = 20)	Minas Gerais (Brazil)	Natural	↑ IgM in all infected dogs.	[36]
			L.i. antigen (polyclonal)	AD (*n* = 2)<br>SD (*n* = 4)	St Feliu de Codines (Spain)	Experimental	IgM appeared in SD later in the infection.	[7]

				CD (*n* = 20)				
			L.i. antigen (polyclonal)	AD (*n* = 132)<br>SD (*n* = 106)<br>CD (*n* = 20)	Barcelona and Mallorca (Spain)	Natural	IgM associated with SD.	[8]
			SLA (polyclonal)	AD-I (*n* = 8)<br>AD-II (*n* = 10)<br>SD (*n* = 16)<br>CD (*n* = 7)	Belo Horizonte (Brazil)	Natural	IgM ↑ in AD-II and SD.	[42]
			SLA (polyclonal)	AD (*n* = 10)<br>SD (*n* = 68)<br>CD (*n* = 7)	Fortaleza (Brazil)	Natural	IgM associated with SD.	[44]
**IgA**	-	ELISA	*L.i.* antigen (polyclonal)	AD (*n* = 2)<br>SD (*n* = 4)<br>CD (*n* = 20)	St Feliu de Codines (Spain)	Experimental	IgA ↑ with time in SD.	[7]
			L.i. antigen (polyclonal)	AD (*n* = 132)<br>SD (*n* = 106)<br>CD (*n* = 20)	Barcelona and Mallorca (Spain)	Natural	IgA associated with SD.	[8]
			SLA (polyclonal)	AD (*n* = 12)<br>OD (*n* = 12)<br>SD (*n* = 16)<br>CD (*n* = 20)	Minas Gerais (Brazil)	Natural	IgA associated to SD.	[36]
			SLA (polyclonal)	AD-I (*n* = 8)<br>AD-II (*n* = 10)<br>SD (*n* = 16)<br>CD (*n* = 7)	Belo Horizonte (Brazil)	Natural	IgA ↑ in AD-II and SD.	[42]
			SLA (polyclonal)	AD (*n* = 10)<br>SD (*n* = 68)<br>CD (*n* = 7)	Fortaleza (Brazil)	Natural	IgA ↑ in infected dogs.	[44]
**IgE**	-	ELISA	f/t *L.i.* antigen (polyclonal)	AD (*n* = 13<br>SD (*n* = 42)<br>CDE (*n* = 21)<br>CDNE (*n* = 18)	E and NE areas from Brazil	Natural	IgE associated with SD.	[34]
			Protein A (polyclonal)	AD (*n* = 29)<br>SD (*n* = 23)<br>CDE (*n* = 3)<br>CDNE (*n* = 19)	Spain & France	Natural	IgE associated with SD.	[35]
			SLA (polyclonal)	AD (*n* = 12)<br>OD (*n* = 12)<br>SD (*n* = 16)<br>CD (*n* = 20)	Minas Gerais (Brazil)	Natural	IgE associated to SD.	[36]
			SLA (polyclonal)	AD-I (*n* = 8)<br>AD-II (*n* = 10)<br>SD (*n* = 16)<br>CD (*n* = 7)	Belo Horizonte (Brazil)	Natural	IgE associated with SD.	[42]
			SLA (polyclonal)	AD (*n* = 10)<br>SD (*n* = 68)<br>CD (*n* = 7)	Fortaleza (Brazil)	Natural	IgE is not correlated with clinical signs.	[44]
			CLA or SLA (polyclonal)	AD$^+$ (*n* = 21)<br>AD$^-$ (*n* = 62)<br>SD$^+$ (*n* = 52)<br>SD$^-$ (*n* = 25)	Araçatuba (Brazil)	Natural	IgE ↑ in all infected dogs.	[45]

CLA: crude *Leishmania* antigen; f/t antigen: freezed/thawed antigen; SLA: soluble *Leishmania* antigen; AD: asymptomatic dogs; AD-I; asymptomatic dogs with negative serology; AD-II: asymptomatic dogs with positive serology ; OD: oligosymptomatic dogs; PD: polysymptomatic dogs; SD: symptomatic dogs; TSD: treated symptomatic dogs; TD: treated infected dogs; CD: control healthy dogs; CDE: control healthy dogs from endemic areas; CDNE: control healthy dogs from non-endemic areas; VD: vaccinated healthy dogs; AD$^+$: asymptomatic dogs infected with *L. chagasi*; AD$^-$: asymptomatic dogs uninfected with *L. chagasi*; SD$^+$: symptomatic dogs infected with *L. chagasi*; SD$^-$: symptomatic dogs uninfected with *L. chagasi*; E: endemic areas; NE: non-endemic areas; pAb: polyclonal antibodies; mAb: monoclonal antibodies; *L.i.*: *Leishmania infantum*; *L.c.*: *Leishmania chagasi*; *L.b.*: *Leishmania braziliensis*.

References

1. Pinelli, E.; Killick-Kendrick, R.; Wagenaar, J.; Bernadina, W.; Del Real, G.; Ruitenberg, J. Cellular and humoral immune responses in dogs experimentally and naturally infected with *Leishmania infantum*. *Infect. Immun.* **1994**, *62*, 229–235, doi:10.1128/iai.62.1.229-235.1994.
2. Leandro, C.; Santos-Gomes, G.M.; Campino, L.; Romão, P.; Cortes, S.; Rolão, N.; Gomes-Pereira, S.; Riça Capela, M.J.; Abranches, P. Cell mediated immunity and specific IgG1 and IgG2 antibody response in natural and experimental canine

leishmaniosis. *Vet. Immunol. Immunopathol.* **2001**, *79*, 273–284, doi:10.1016/S0165-2427(01)00270-7.
3. Fernández-Pérez, F.J.; Gómez-Muñoz, M.T.; Méndez, S.; Alunda, J.M. Leishmania-specific lymphoproliferative responses and IgG1/IgG2 immunodetection patterns by Western blot in asymptomatic, symptomatic and treated dogs. *Acta Trop.* **2003**, *86*, 83–91, doi:10.1016/S0001-706X(03)00004-4.
4. Carrillo, E.; Ahmed, S.; Goldsmith-Pestana, K.; Nieto, J.; Osorio, Y.; Travi, B.; Moreno, J.; McMahon-Pratt, D. Immunogenicity of the P-8 amastigote antigen in the experimental model of canine visceral leishmaniasis. *Vaccine* **2007**, *25*, 1534–1543, doi:10.1016/j.vaccine.2006.10.036.
5. Carrillo, E.; Crusat, M.; Nieto, J.; Chicharro, C.; Thomas, M. del C.; Martínez, E.; Valladares, B.; Cañavate, C.; Requena, J.M.; López, M.C.; et al. Immunogenicity of HSP-70, KMP-11 and PFR-2 leishmanial antigens in the experimental model of canine visceral leishmaniasis. *Vaccine* **2008**, *26*, 1902–1911, doi:10.1016/j.vaccine.2008.01.042.
6. De Luna, R.; Vuotto, M.L.; Ielpo, M.T.L.; Ambrosio, R.; Piantedosi, D.; Moscatiello, V.; Ciaramella, P.; Scalone, A.; Gradoni, L.; Mancino, D. Early suppression of lymphoproliferative response in dogs with natural infection by *Leishmania infantum*. *Vet. Immunol. Immunopathol.* **1999**, *70*, 95–103, doi:10.1016/S0165-2427(99)00073-2.
7. Rodríguez-Cortés, A.; Ojeda, A.; López-Fuertes, L.; Timón, M.; Altet, L.; Solano-Gallego, L.; Sánchez-Robert, E.; Francino, O.; Alberola, J. A long term experimental study of canine visceral leishmaniasis. *Int. J. Parasitol.* **2007**, *37*, 683–693, doi:10.1016/j.ijpara.2006.11.007.
8. Rodríguez-Cortés, A.; Fernández-Bellón, H.; Ramis, A.; Ferrer, L.; Alberola, J.; Solano-Gallego, L. Leishmania-specific isotype levels and their relationship with specific cell-mediated immunity parameters in canine leishmaniasis. *Vet. Immunol. Immunopathol.* **2007**, *116*, 190–198, doi:10.1016/j.vetimm.2007.01.015.
9. Travi, B.L.; Osorio, E.Y.; Saldarriaga, O.A.; Cadena, H.; Tabares, C.J.; Peniche, A.; Lee, S.; Melby, P.C. Clinical, parasitologic, and immunologic evolution in dogs experimentally infected with sand fly-derived *Leishmania chagasi* promastigotes. *Am. J. Trop. Med. Hyg.* **2009**, *81*, 994–1003, doi:10.4269/ajtmh.2009.09-0229.
10. Da Costa Pinheiro, P.H.; De Souza Dias, S.; Dantas Eulálio, K.; Mendonça, I.L.; Katz, S.; Barbiéri, C.L. Recombinant cysteine proteinase from Leishmania (Leishmania) chagasi implicated in human and dog T-cell responses. *Infect. Immun.* **2005**, *73*, 3787–3789, doi:10.1128/IAI.73.6.3787-3789.2005.
11. Rhalem, A.; Sahibi, H.; Guessous-Idrissi, N.; Lasri, S.; Natami, A.; Riyad, M.; Berrag, B. Immune response against Leishmania antigens in dogs naturally and experimentally infected with *Leishmania infantum*. *Vet. Parasitol.* **1999**, *81*, 173–184, doi:10.1016/S0304-4017(98)00240-4.
12. Rafati, S.; Nakhaee, A.; Taheri, T.; Ghashghaii, A.; Salmanian, A.H.; Jimenez, M.; Mohebali, M.; Masina, S.; Fasel, N. Expression of cysteine proteinase type I and II of *Leishmania infantum* and their recognition by sera during canine and human visceral leishmaniasis. *Exp. Parasitol.* **2003**, *103*, 143–151, doi:10.1016/S0014-4894(03)00097-3.
13. Nakhaee, A.; Taheri, T.; Taghikhani, M.; Mohebali, M.; Salmanian, A.H.; Fasel, N.; Rafati, S. Humoral and cellular immune responses against Type I cysteine proteinase of *Leishmania infantum* are higher in asymptomatic than symptomatic dogs selected from a naturally infected population. *Vet. Parasitol.* **2004**, *119*, 107–123, doi:10.1016/j.vetpar.2003.11.013.
14. Boggiatto, P.M.; Ramer-Tait, A.E.; Metz, K.; Kramer, E.E.; Gibson-Corley, K.; Mullin, K.; Hostetter, J.M.; Gallup, J.M.; Jones, D.E.; Petersen, C.A. Immunologic indicators of clinical progression during canine *Leishmania infantum* infection. *Clin. Vaccine Immunol.* **2010**, *17*, 267–273, doi:10.1128/CVI.00456-09.
15. Esch, K.J.; Juelsgaard, R.; Martinez, P.A.; Jones, D.E.; Petersen, C.A. Programmed Death 1–Mediated T Cell Exhaustion during Visceral Leishmaniasis Impairs Phagocyte Function. *J. Immunol.* **2013**, *191*, 5542–5550, doi:10.4049/jimmunol.1301810.
16. Cabral, M.; O'Grady, J.; Alexander, J. Demonstration of Leishmania specific cell mediated and humoral immunity in asymptomatic dogs. *Parasite Immunol.* **1992**, *14*, 531–539, doi:10.1111/j.1365-3024.1992.tb00026.x.
17. Cabral, M.; O'Grady, J.E.; Gomes, S.; Sousa, J.C.; Thompson, H.; Alexander, J. The immunology of canine leishmaniosis: Strong evidence for a developing disease spectrum from asymptomatic dogs. *Vet. Parasitol.* **1998**, *76*, 173–180, doi:10.1016/S0304-4017(97)00208-2.
18. Pinelli, E.; Gonzalo, R.M.; Boog, C.J.P.; Rutten, V.P.M.G.; Gebhard, D.; Del Real, G.; Ruitenberg, E.J. *Leishmania infantum*-specific T cell lines derived from asymptomatic dogs that lyse infected macrophages in a major histocompatibility complex-restricted manner. *Eur. J. Immunol.* **1995**, *25*, 1594–1600, doi:10.1002/eji.1830250619.
19. Schaut, R.G.; Lamb, I.M.; Toepp, A.J.; Scott, B.; Mendes-Aguiar, C.O.; Coutinho, J.F. V.; Jeronimo, S.M.B.; Wilson, M.E.; Harty, J.T.; Waldschmidt, T.J.; et al. Regulatory IgD hi B Cells Suppress T Cell Function via IL-10 and PD-L1 during Progressive Visceral Leishmaniasis . *J. Immunol.* **2016**, *196*, 4100–4109, doi:10.4049/jimmunol.1502678.
20. Cortese, L.; Annunziatella, M.; Palatucci, A.T.; Rubino, V.; Piantedosi, D.; Di Loria, A.; Ruggiero, G.; Ciaramella, P.; Terrazzano, G. Regulatory T cells, Cytotoxic T lymphocytes and a Th1 cytokine profile in dogs naturally infected by *Leishmania infantum*. *Res. Vet. Sci.* **2013**, *95*, 942–949, doi:10.1016/j.rvsc.2013.08.005.
21. Gomes de Almeida Leal, G.; Mendes Roatt, B.; Dian de Oliveira Aguiar-Soares, R.; Martins Carneiro, C.; Cordeiro Giunchetti, R.; Teixeira-Carvalho, A.; Assis Martins-Filho, O.; Fortes Francisco, A.; Mirelle Cardoso, J.; Siqueira Mathias, F.A.; et al. Immunological profile of resistance and susceptibility in naturally infected dogs by *Leishmania infantum*. *Vet. Parasitol.* **2014**, *205*, 472–482, doi:10.1016/j.vetpar.2014.08.022.
22. Matralis, D.; Papadogiannakis, E.; Kontos, V.; Papadopoulos, E.; Ktenas, E.; Koutinas, A. Detection of intracellular IFN-γ and IL-4 cytokines in CD4+ and CD8+ T cells in the peripheral blood of dogs naturally infected with *Leishmania infantum*. *Parasite Immunol.* **2016**, *38*, 510–515, doi:10.1111/pim.12335.
23. Solano-Gallego, L.; Montserrat-Sangrà, S.; Ordeix, L.; Martínez-Orellana, P. *Leishmania infantum*-specific production of IFN-γ and IL-10 in stimulated blood from dogs with clinical leishmaniosis. *Parasites and Vectors* **2016**, *9*, 1–10, doi:10.1186/s13071-016-

1598-y.
24. Panaro, M.A.; Brandonisio, O.; Cianciulli, A.; Cavallo, P.; Lacasella, V.; Paradies, P.; Testini, G.; De Caprariis, D.; Mitolo, V.; Otranto, D. Cytokine expression in dogs with natural *Leishmania infantum* infection. *Parasitology* **2009**, *136*, 823–831, doi:10.1017/S0031182009006155.
25. Chamizo, C.; Moreno, J.; Alvar, J. Semi-quantitative analysis of cytokine expression in asymptomatic canine leishmaniasis. *Vet. Immunol. Immunopathol.* **2005**, *103*, 67–75, doi:10.1016/j.vetimm.2004.08.010.
26. Solcà, M.S.; Andrade, B.B.; Abbehusen, M.M.C.; Teixeira, C.R.; Khouri, R.; Valenzuela, J.G.; Kamhawi, S.; Bozza, P.T.; Fraga, D.B.M.; Borges, V.M.; et al. Circulating Biomarkers of Immune Activation, Oxidative Stress and Inflammation Characterize Severe Canine Visceral Leishmaniasis. *Sci. Rep.* **2016**, *6*, 32619:1-32619:7, doi:10.1038/srep32619.
27. Abbehusen, M.M.C.; Dos Anjos Almeida, V.; Da, S.M.; Da Silva Pereira, L.; Costa, D.J.; Gil-Santana, L.; Bozza, P.T.; Fraga, D.B.M.; Veras, P.S.T.; Dos-Santos, W.L.C.; et al. Clinical and immunopathological findings during long term follow-up in *Leishmania infantum* experimentally infected dogs. *Sci. Rep.* **2017**, *7*, 1–11, doi:10.1038/s41598-017-15651-8.
28. Barbosa, M.A.G.; Alexandre-Pires, G.; Soares-Clemente, M.; Marques, C.; Rodrigues, O.R.; De Brito, T.V.; Da Fonseca, I.P.; Alves, L.C.; Santos-Gomes, G.M. Cytokine Gene Expression in the Tissues of Dogs Infected by *Leishmania infantum*. *J. Comp. Pathol.* **2011**, *145*, 336–344, doi:10.1016/j.jcpa.2011.03.001.
29. Manna, L.; Reale, S.; Viola, E.; Vitale, F.; Manzillo, V.F.; Michele, P.L.; Caracappa, S.; Gravino, A.E. Leishmania DNA load and cytokine expression levels in asymptomatic naturally infected dogs. *Vet. Parasitol.* **2006**, *142*, 271–280, doi:10.1016/j.vetpar.2006.06.028.
30. Bourdoiseau, G.; Bonnefont, C.; Hoareau, E.; Boehringer, C.; Stolle, T.; Chabanne, L. Specific IgG1 and IgG2 antibody and lymphocyte subset levels in naturally *Leishmania infantum*-infected treated and untreated dogs. *Vet. Immunol. Immunopathol.* **1997**, *59*, 21–30, doi:10.1016/S0165-2427(97)00072-X.
31. Nieto, C.G.; García-Alonso, M.; Requena, J.M.; Mirón, C.; Soto, M.; Alonso, C.; Navarrete, I. Analysis of the humoral immune response against total and recombinant antigens of *Leishmania infantum*: Correlation with disease progression in canine experimental leishmaniasis. *Vet. Immunol. Immunopathol.* **1999**, *67*, 117–130, doi:10.1016/S0165-2427(98)00213-X.
32. Solano-Gallego, L.; Riera, C.; Roura, X.; Iniesta, L.; Gallego, M.; Valladares, J.E.; Fisa, R.; Castillejo, S.; Alberola, J.; Ferrer, L.; et al. *Leishmania infantum*-specific IgG, IgG1 and IgG2 antibody responses in healthy and ill dogs from endemic areas: Evolution in the course of infection and after treatment. *Vet. Parasitol.* **2001**, *96*, 265–276, doi:10.1016/S0304-4017(00)00446-5.
33. Cordeiro-da-Silva, A.; Cardoso, L.; Araújo, N.; Castro, H.; Tomás, A.; Rodrigues, M.; Cabral, M.; Vergnes, B.; Sereno, D.; Ouaissi, A. Identification of antibodies to Leishmania silent information regulatory 2 (SIR2) protein homologue during canine natural infections: Pathological implications. *Immunol. Lett.* **2003**, *86*, 155–162, doi:10.1016/S0165-2478(03)00020-8.
34. Almeida, M.A.O.; Jesus, E.E.V.; Sousa-Atta, M.L.B.; Alves, L.C.; Berne, M.E.A.; Atta, A.M. Antileishmanial antibody profile in dogs naturally infected with *Leishmania chagasi*. *Vet. Immunol. Immunopathol.* **2005**, *106*, 151–158, doi:10.1016/j.vetimm.2004.08.024.
35. Iniesta, L.; Gállego, M.; Portús, M. Immunoglobulin G and E responses in various stages of canine leishmaniosis. *Vet. Immunol. Immunopathol.* **2005**, *103*, 77–81, doi:10.1016/j.vetimm.2004.08.011.
36. Reis, A.B.; Teixeira-Carvalho, A.; Vale, A.M.; Marques, M.J.; Giunchetti, R.C.; Mayrink, W.; Guerra, L.L.; Andrade, R.A.; Corrêa-Oliveira, R.; Martins-Filho, O.A. Isotype patterns of immunoglobulins: Hallmarks for clinical status and tissue parasite density in brazilian dogs naturally infected by Leishmania (Leishmania) chagasi. *Vet. Immunol. Immunopathol.* **2006**, *112*, 102–116, doi:10.1016/j.vetimm.2006.02.001.
37. Cardoso, L.; Schallig, H.D.F.H.; Cordeiro-da-Silva, A.; Cabral, M.; Alunda, J.M.; Rodrigues, M. Anti-Leishmania humoral and cellular immune responses in naturally infected symptomatic and asymptomatic dogs. *Vet. Immunol. Immunopathol.* **2007**, *117*, 35–41, doi:10.1016/j.vetimm.2007.01.014.
38. da Costa-Val, A.P.; Cavalcanti, R.R.; de Figueiredo Gontijo, N.; Marques Michalick, M.S.; Alexander, B.; Williams, P.; Melo, M.N. Canine visceral leishmaniasis: Relationships between clinical status, humoral immune response, haematology and Lutzomyia (Lutzomyia) longipalpis infectivity. *Vet. J.* **2007**, *174*, 636–643, doi:10.1016/j.tvjl.2006.11.006.
39. Iniesta, L.; Gállego, M.; Portús, M. Idiotype expression of IgG1 and IgG2 in dogs naturally infected with *Leishmania infantum*. *Vet. Immunol. Immunopathol.* **2007**, *119*, 189–197, doi:10.1016/j.vetimm.2007.05.006.
40. Carson, C.; Quinnell, R.J.; Day, M.J.; Courtenay, O. Comparison of monoclonal and polyclonal antibodies for the detection of canine IgG1 and IgG2, and associations with infection outcome in *Leishmania infantum* naturally infected dogs. *Vet. Immunol. Immunopathol.* **2010**, *133*, 264–268, doi:10.1016/j.vetimm.2009.07.017.
41. Neto, R.G.T.; Giunchetti, R.C.; Carneiro, C.M.; Vitor, R.W. de A.; Coura-Vital, W.; Quaresma, P.F.; Ker, H.G.; Melo, L.A. de; Gontijo, C.M.F.; Reis, A.B. Relationship of Leishmania-specific IgG levels and IgG avidity with parasite density and clinical signs in canine leishmaniasis. *Vet. Parasitol.* **2010**, *169*, 248–257, doi:10.1016/j.vetpar.2010.01.023.
42. Coura-Vital, W.; Marques, M.J.; Giunchetti, R.C.; Teixeira-Carvalho, A.; Moreira, N.D.; Vitoriano-Souza, J.; Vieira, P.M.; Carneiro, C.M.; Corrêa-Oliveira, R.; Martins-Filho, O.A.; et al. Humoral and cellular immune responses in dogs with inapparent natural *Leishmania infantum* infection. *Vet. J.* **2011**, *190*, 43–47, doi:10.1016/j.tvjl.2011.04.005.
43. Ribeiro, F.C.; Schubach, A. de O.; Mouta-Confort, E.; Pacheco, T.M.V.; Madeira, M. de F.; Abboud, L.C. de S.; Honse, C. de O.; Alves, A.S.; Marzochi, M.C.A. Use of ELISA employing homologous and heterologous antigens for the dectection of IgG and subclasses (IgG1 and IgG2) in the diagnosis of Canine Visceral Leishmaniasis. *Rev. Inst. Med. Trop. Sao Paulo* **2011**, *53*, 283–289, doi:10.1590/S0036-46652011000500008.
44. De Freitas, J.C.C.; Lopes-Neto, B.E.; De Abreu, C.R.A.; Coura-Vital, W.; Braga, S.L.; Reis, A.B.; Nunes-Pinheiro, D.C.S. Profile of anti-Leishmania antibodies related to clinical picture in canine visceral leishmaniasis. *Res. Vet. Sci.* **2012**, *93*, 705–709,

doi:10.1016/j.rvsc.2011.12.009.
45. Laranjeira, D.F.; Da Matta, V.L.R.; Tomokane, T.Y.; Marcondes, M.; Corbet, C.E.P.; Laurenti, M.D. Serological and infection statuses of dogs from a visceral leishmaniasis-endemic area. *Rev. Saude Publica* **2014**, *48*, 563–570, doi:10.1590/S0034-8910.2014048005224.
46. Lima, L.V.D.R.; Carneiro, L.A.; Campos, M.B.; Vasconcelos Dos Santos, T.; Ramos, P.K.; Laurenti, M.D.; Teixeira, C.E.C.; Silveira, F.T. Further evidence associating IgG1, but not IgG2, with susceptibility to canine visceral leishmaniasis caused by Leishmania (L.) infantum chagasi -infection. *Parasite* **2017**, *24*, 37:1-37:11, doi:10.1051/parasite/2017039.
47. Chaabouni, A.; Boubaker Elandoulsi, R.; Mhadhbi, M.; Gharbi, M.; Sassi, A. Comparative analysis of the *Leishmania infantum*-specific antibody repertoires and the autoantibody repertoires between asymptomatic and symptomatic dogs. *Vet. Parasitol.* **2018**, *261*, 9–17, doi:10.1016/j.vetpar.2018.07.011.
48. Campos, M.P.; Figueiredo, F.B.; Morgado, F.N.; Renzetti, A.R. dos S.; de Souza, S.M.M.; Pereira, S.A.; Rodrigues-Da-Silva, R.N.; Lima-Junior, J.D.C.; De Luca, P.M. *Leishmania infantum* virulence factor A2 protein: Linear B-cell epitope mapping and identification of three main linear B-cell epitopes in vaccinated and naturally infected dogs. *Front. Immunol.* **2018**, *9*, 1690:1-1690:11, doi:10.3389/fimmu.2018.01690.
49. Maia, A.C.R.G.; Porcino, G.N.; Faria-Pinto, P.; Mendes, T.V.; Antinarelli, L.M.R.; Coimbra, E.S.; Reis, A.B.; Juliano, L.; Juliano, M.A.; Marques, M.J.; et al. *Leishmania infantum* nucleoside triphosphate diphosphohydrolase 1 (NTPDase 1) B-domain: Antibody antiproliferative effect on the promastigotes and IgG subclass responses in canine visceral leishmaniasis. *Vet. Parasitol.* **2019**, *271*, 38–44, doi:10.1016/j.vetpar.2019.06.004.
50. Agallou, M.; Athanasiou, E.; Samiotaki, M.; Panayotou, G.; Karagouni, E. Identification of immunoreactive *Leishmania infantum* protein antigens to asymptomatic dog sera through combined immunoproteomics and bioinformatics analysis. *PLoS One* **2016**, *11*, 1–22, doi:10.1371/journal.pone.0149894.
51. Quinnell, R.J.; Courtenay, O.; Garcez, L.M.; Kaye, P.M.; Shaw, M.A.; Dye, C.; Day, M.J. IgG subclass responses in a longitudinal study of canine visceral leishmaniasis. *Vet. Immunol. Immunopathol.* **2003**, *91*, 161–168, doi:10.1016/S0165-2427(02)00311-2.
52. Oliveira, T.M.F.S.; Mineo, T.W.P.; Bason, M.; Day, M.J.; Machado, R.Z. IgG subclass profile of serum antibodies to *Leishmania chagasi* in naturally infected and vaccinated dogs. *Vet. Parasitol.* **2009**, *162*, 16–22, doi:10.1016/j.vetpar.2009.02.018.
53. Marcondes, M.; Ikeda, F.A.; Vieira, R.F.C.; Day, M.J.; Lima, V.M.F.; Rossi, C.N.; Perri, S.H.V.; Biondo, A.W. Temporal IgG subclasses response in dogs following vaccination against *Leishmania* with Leishmune ®. *Vet. Parasitol.* **2011**, *181*, 153–159, doi:10.1016/j.vetpar.2011.04.004.
